# Initiating Resuscitation Before Umbilical Cord Clamping in Infants with Congenital Diaphragmatic Hernia: A Pilot Feasibility Trial

**DOI:** 10.1136/archdischild-2019-317477

**Published:** 2019-08-28

**Authors:** Elizabeth E. Foglia, Anne Ades, Holly L. Hedrick, Natalie Rintoul, David Munson, Julie S. Moldenhauer, Juliana Gebb, Bonnie Serletti, Aasma Chaudhary, Danielle D. Weinberg, Natalie Napolitano, María V. Fraga, Sarah J. Ratcliffe

**Affiliations:** 1 Children’s Hospital of Philadelphia, Philadelphia PA; 2 Division of Biostatistics, Department of Public Health Sciences, University of Virginia, Charlottesville VA

## Abstract

**Background:**

Infants with congenital diaphragmatic hernia (CDH) often experience hypoxemia with acidosis immediately after birth. The traditional approach in the delivery room is immediate cord clamping followed by intubation. Initiating resuscitation prior to umbilical cord clamping (UCC) may support this transition.

**Objectives:**

To establish the safety and feasibility of intubation and ventilation prior to UCC for infants with CDH. To compare short-term outcomes between trial participants and matched controls treated with immediate cord clamping before intubation and ventilation.

**Design:**

Single-arm, single site trial of infants with CDH and gestational age ≥ 36 weeks. Infants were placed on a trolley immediately after birth and underwent intubation and ventilation, with UCC performed after qualitative CO_2_ detection. The primary feasibility endpoint was successful intubation prior to UCC. Prespecified safety and physiologic outcomes were compared with historical controls matched for prognostic variables using standard bivariate tests.

**Results:**

Of 20 enrolled infants, all were placed on the trolley and 17 (85%) were intubated before UCC. The first hemoglobin and mean blood pressure at 1 hour of life were significantly higher in trial participants than controls. There were no significant differences between groups for subsequent blood pressure values, vasoactive medications, inhaled nitric oxide, or ECMO. Blood gas and oxygenation index values did not differ between groups at any point.

**Conclusions:**

Intubation and ventilation prior to UCC is safe and feasible among infants with CDH. The impact of this approach on clinically relevant outcomes deserves investigation in a randomized trial.

## Introduction:

Physiologic adaptation after birth is a critical transition for infants with congenital diaphragmatic hernia (CDH). In utero, the fetus has fluid-filled lungs, and gas exchange occurs at the placenta. Immediately after birth, the infant must aerate the lungs, which triggers a physiologic transition of reduced pulmonary vascular resistance, increased pulmonary blood flow, and gas exchange in the lung. (1,2) Infants with CDH struggle to independently achieve lung aeration due to pulmonary hypoplasia and space-occupying effects of herniated abdominal organs. Further, they are at risk for decreased pulmonary blood flow due to pulmonary hypertension. In a preclinical model of CDH, physiologic challenges during transition include lower lung compliance, more respiratory acidosis, and poor cerebral oxygenation.(3) Clinically this manifests as hypoxemia, hypercarbia, and acidosis after birth, often reflected in low Apgar scores.(4–6)

Limited data inform the delivery room management for infants with CDH.(7) The standard approach is immediate umbilical cord clamping (UCC) followed by intubation and ventilation.(8–10) An alternative strategy is to establish lung aeration prior to UCC, which has been called “physiologically based cord clamping.”(11) Physiologically based cord clamping may stabilize gas exchange during neonatal transition among infants with CDH by supporting aeration of the hypoplastic lung and increasing pulmonary blood flow through the thickened pulmonary vasculature before UCC. In an ovine CDH model, ventilation onset before UCC resulted in improved cerebral tissue oxygenation and increased pulmonary blood flow compared with UCC before ventilation.(12) A physiologically based approach to UCC for infants with CDH may smooth the postnatal transition and prevent acidosis and hypoxemia.

Performing intubation and establishing ventilation prior to UCC in the delivery room entails coordination of multiple team members and equipment. Previous investigators found that initiating non-invasive respiratory support prior to UCC was safe and feasible in other populations, (13–18) but few infants in those studies required invasive support. One small before/after study demonstrated the feasibility of performing intubation and initiating ventilation before UCC among infants with CDH, but most infants in that study had mild CDH prognostic parameters.(19)

The objectives of this pilot trial were to: 1) establish the safety and feasibility of intubation and ventilation prior to UCC for infants with CDH and 2) compare short-term outcomes between infants treated with intubation and ventilation prior to UCC and matched historical controls treated with immediate cord clamping before intubation and ventilation.

## Methods:

### Study Design and Setting

This was a single-arm, single site trial at the Children’s Hospital of Philadelphia (Clinicaltrials.gov Identifier NCT03314233). Eligible infants had an antenatal diagnosis of CDH and were at least 36 weeks gestation at birth. Exclusion criteria were: other major anomalies, enrollment in an ongoing trial of Fetoscopic Endoluminal Tracheal Occlusion (FETO), multiple gestation, palliative care planned or considered, placental abnormalities (abruption, previa, or accreta), and maternal magnesium sulfate therapy. Informed consent was obtained from mothers of potentially eligible infants in the prenatal clinic setting, and eligibility criteria were confirmed immediately before delivery. If all eligibility criteria were met, the mother/infant dyad was allocated to receive the study intervention.

### Intervention

**Figure 1:**
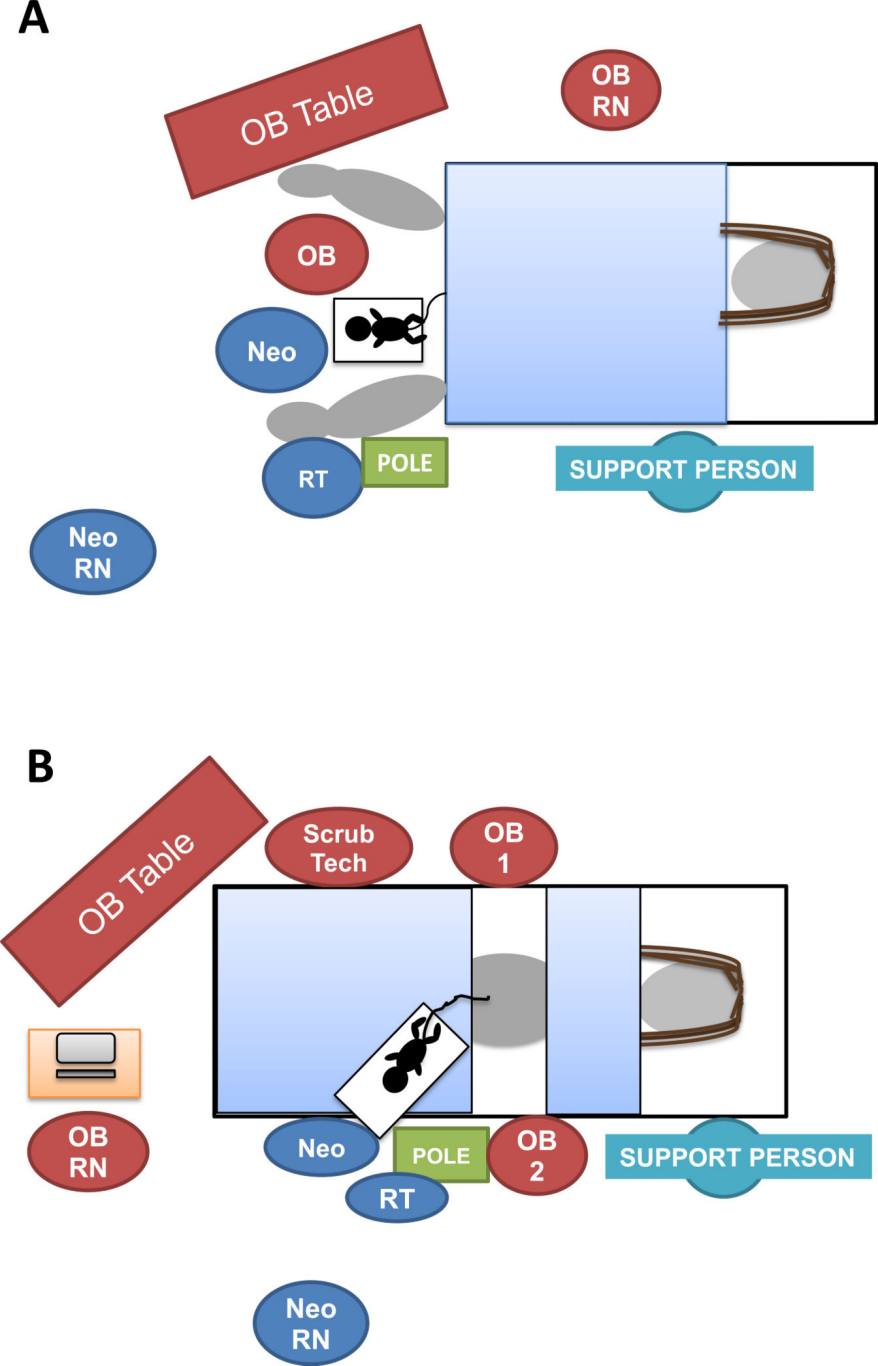
Team and equipment positioning for (A) vaginal deliveries and (B) cesarean deliveries Neo: neonatologist, OB: obstetrical/obstetrician, Pole: mobile respiratory pole with T-piece resuscitator, RN: nurse, RT: respiratory therapist

An attending neonatologist, respiratory therapist, and neonatal nurse attended each delivery (Figure 1). A mobile LifeStart Trolley (Inditherm Medical, Rotherham United Kingdom) was adjusted to the level of the introitus for vaginal deliveries and just above the level of the incision for cesarean deliveries. An activated chemical warmer mattress was placed on the trolley mattress for thermoregulation. For cesarean deliveries, the trolley and neonatal providers were sterilely draped.

Initially, the study used a purpose-built respiratory pole with air and oxygen tanks and a Neopuff Infant T-piece resuscitator (Fisher and Paykel, Auckland New Zealand). Suction tubing was connected to canisters in the labor room or obstetrical suite. Midway through the trial, this equipment was replaced by a portable T-piece resuscitator that included suction (Giraffe Stand Alone Resuscitation System, GE Healthcare, USA). Airway equipment and respiratory tubing were not sterile and were handled to avoid contact with the sterile operative field for cesarean deliveries.

Immediately after delivery, the obstetrical provider placed the infant on the trolley. The neonatologist intubated the infant, and ventilation was commenced using settings per hospital protocol (initial pressures 20–25/5 cm H_2_O, FiO_2_ 0.5). UCC was performed after consistent qualitative colorimetric CO_2_ detection, with guidelines for maternal uterotonic administration after UCC. After UCC, the neonatal team moved the trolley and respiratory equipment away from the mother, taped the endotracheal tube, covered the infant with warm towels, and transported the infant with ongoing ventilation to the resuscitation warmer bed. All remaining care was per clinical guidelines. The protocol stipulated for UCC to be performed prior to intubation for any of the following: the infant could not be placed on the trolley, intubation was not successful within 3 minutes from birth, cord avulsion or bleeding, or any obstetrical or neonatal provider concerns.

The study team developed training videos and conducted equipment and simulation training for staff. A study team member was present for all resuscitations to ensure protocol adherence. When time allowed, the intervention was video recorded.

### Outcomes:

The primary feasibility endpoint was successful intubation prior to UCC within the 3 minute timeframe. Prespecified neonatal safety outcomes included cord avulsion, cardiopulmonary resuscitation, and first measured temperature. Maternal safety outcomes included estimated blood loss, therapeutic uterotonics, and obstetrical concern for contamination of the sterile surgical field and wound infection (for cesarean deliveries). Feasibility outcomes and timing of procedural interventions were recorded by a dedicated staff member and abstracted from video recordings when available.

Physiologic outcomes included first hemoglobin measurement, mean blood pressure values and blood gas parameters in the first 48 hours after birth, the first clinically obtained echocardiogram, and mortality in the first week after birth. Echocardiogram images were independently reviewed by an unblinded single assessor (MF) for signs of pulmonary hypertension. If present, the severity of pulmonary hypertension was graded based on the most severe assessment of the following criteria: direction of shunting at the ductus arteriosus (bidirectional: moderate; all right to left: severe), estimated systolic right ventricular pressures through continuous wave interrogation of the tricuspid regurgitant jet and calculation of a systolic right ventricle-to-right atrium pressure gradient by using the modified Bernoulli equation [pressure gradient = 4 × Jet velocity^2^](< ½ systemic: normal/mild; >= ½ and <systemic: moderate; >= systemic: severe) and qualitative evaluation of interventricular septal position at the end of systole (rounded: normal/mild, flattened: moderate, bowed: severe).

Interventions assessed included respiratory support and inhaled nitric oxide in the first 48 hours of life, and extracorporeal membrane oxygenation (ECMO) in the first week after birth. Historical controls were identified from an ongoing pulmonary hypoplasia program registry and matched with trial participants on the basis of gestational age, mode of delivery, side of CDH, liver position (intrathoracic vs intra-abdominal), and observed to expected lung-to-head ratio (O/E LHR) obtained by anterior-posterior method on prenatal ultrasound using the TOTAL trial calculator.(20) All controls were born after the most recent change to the CDH delivery room clinical protocol in our hospital to ensure consistent management with trial participants outside of the study intervention. (21)

We planned a sample of 20 infants allocated to the intervention for this pilot trial to assess the feasibility of the study procedure. Using Stata 15.1 (StataCorp, College Station, TX), outcomes were reported with descriptive statistics. Safety and physiological outcomes were compared between trial participants and historical controls using t-test for normally distributed variables and Wilcoxon rank sum test for nonparametric variables. Dichotomous variables were assessed with chi-squared test and Fisher’s exact test, as appropriate. A p-value <0.05 was considered statistically significant.

This study was approved by the Children’s Hospital of Philadelphia Institutional Review Board (17-014125). To ensure the trial did not impose undue additional risk on trial participants, an independent Data Safety Monitoring Board reviewed safety outcomes of enrolled participants at three scheduled intervals.

## Results

**Figure 2:**
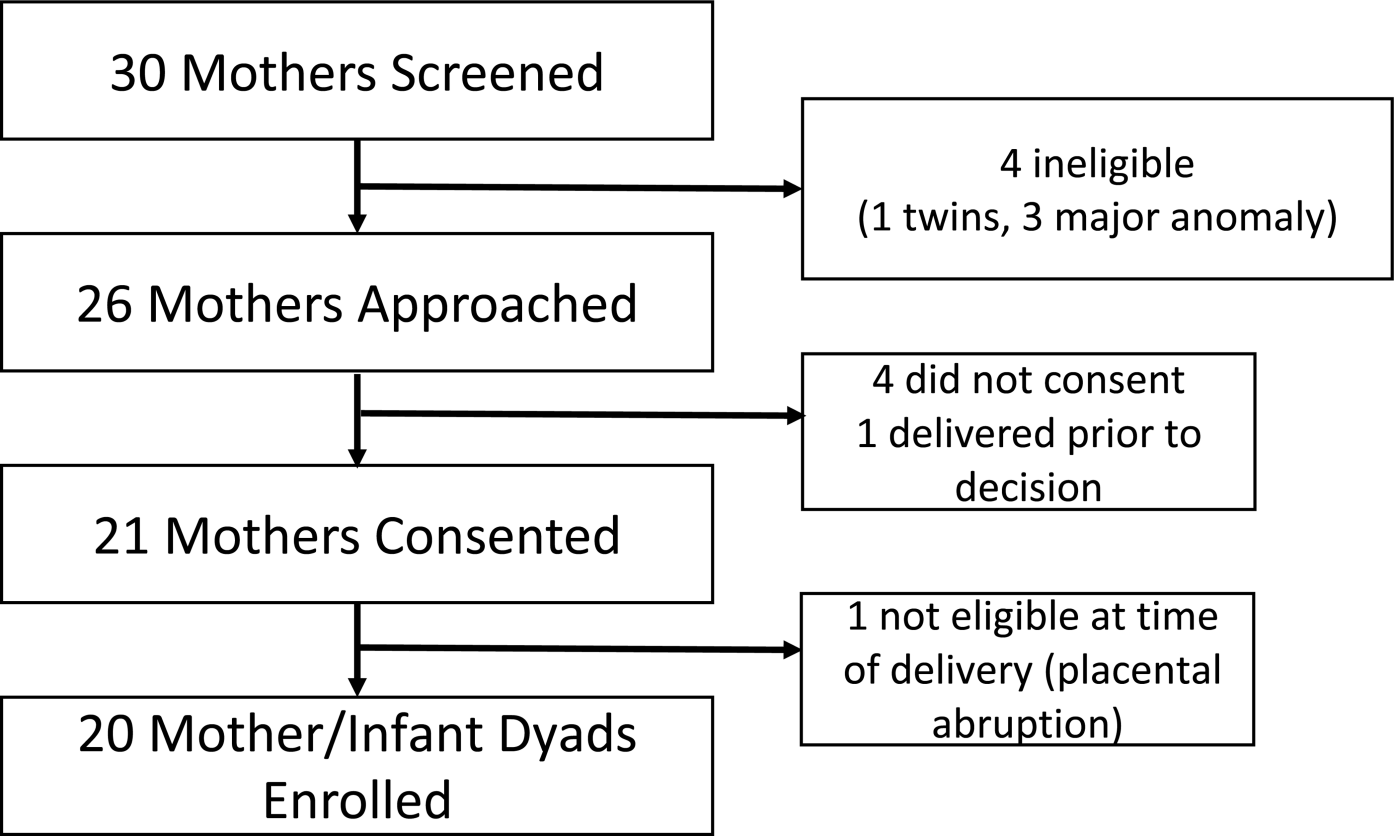
Study flow diagram

Between January-October 2018, 20 eligible infants were allocated to receive the intervention (Figure 2). Demographic characteristics of enrolled infants and matched controls are shown in Table 1. One enrolled infant was diagnosed with a second major anomaly postnatally. This infant was included in the assessment of safety and feasibility but not physiologic outcomes; no control was matched to this participant.

All 20 infants were placed on the trolley and 17 (85%) were successfully intubated prior to UCC (Video, online only). The median interval between birth and UCC was 2 minutes (IQR 1:15, 2:32). Among the 17 successful cases, the median duration between birth and intubation was 1:02 (IQR 00:55, 2:00), with 14 infants intubated on the first attempt and 3 intubated on the second attempt within this timeframe. The median duration between onset of ventilation and colorimetric CO_2_ detection was 14 (IQR 11, 19) seconds. In 3 cases the infant was not successfully intubated by 3 minutes after birth, and the umbilical cord was therefore clamped and cut prior to intubation. Two of these infants were born via vaginal delivery and one via cesarean delivery. Neonatal providers in those cases identified that infant positioning was the major impediment to procedural success, and all 3 of these infants were intubated after UCC once placed on the warmer bed.

Safety outcomes were similar between trial participants and historical controls (Table 2). The first measured hemoglobin was higher among trial participants than historical controls (Table 3). Mean blood pressure values were higher in trial participants at 1 hour of life. Subsequent mean blood pressure values assessed at 6 hours of life and beyond were similar between groups (data not shown). Blood gas parameters and oxygenation indices did not differ between trial participants or controls on the first blood gas or at any point in the first 48 hours after birth. There were no differences between groups in the use of vasoactive medications, inhaled nitric oxide, or ECMO. No trial participants and 1 historical control died in the first week after birth.

Echocardiograms were obtained at a mean of 13 hours after birth. One trial participant and one historical control were supported with ECMO when the echocardiograms were obtained, and these images were not assessed. All infants had evidence of pulmonary hypertension (Supplemental Table).

## Discussion

We conducted this pilot trial to assess the safety and feasibility of initiating resuscitation prior to UCC for infants with CDH. It was possible to position all enrolled infants on a trolley adjacent to their mothers after birth. In most cases, it was possible to perform immediate intubation and ventilation with an intact umbilical cord. We did not find any evidence that this approach to resuscitation poses increased risk to infants or their mothers. Enrolled infants had higher hemoglobin levels and transiently higher systemic blood pressures compared with matched historical controls.

This study contributes to a growing body of literature demonstrating the feasibility and safety of initiating resuscitation prior to UCC. (13–18). In contrast to our study population, few infants in previous trials required invasive respiratory support. In the trial by Duley et al. more invasive interventions were performed during intact-cord resuscitation, including intubation and cardiac resuscitation of enrolled newborns.(15) However, the protocol completion rate was lower among infants in the intervention group in that study (59%) compared with other trials of preterm infants (89–100%). (13,14,16)

Intubation is a standard approach to the delivery room management for all infants with CDH in our hospital. Thus, establishing lung aeration prior to UCC among infants with CDH requires intubation with an intact cord. In this trial, 17/20 infants were intubated within the specified 3 minute timeframe before UCC. The threshold of allowing 3 minutes for intubation prior to UCC was considered to represent a reasonable balance between providing sufficient time for the neonatologist to intubate the infant without introducing an excessive delay in intubation and onset of ventilation if intubation before UCC was not possible. Neonatologists involved in unsuccessful cases expressed that positioning on the trolley was the major impediment to intubation, suggesting that additional time beyond 3 minutes may not have resulted in procedural success.

UCC was performed after colorimetric CO_2_ detection. It is possible that a longer duration of ventilation after intubation would allow for more adequate lung aeration before UCC. Physiologically based cord clamping has been variably defined. Lefebvre et al. specified for UCC to occur after “stabilization,” indicated by measures of heart rate and oxygen saturation. Brouwer et al. targeted vital sign parameters as well as exhaled tidal volume > 4mL/kg as an indicator of lung aeration among preterm infants.(16) This may not be a reasonable target for infants with CDH and related pulmonary hypoplasia, in whom tidal volumes after birth are often <4 mL/kg for both spontaneous breaths and manual inflations.(22,23) In addition, tidal volume monitoring is not standard of care in most delivery rooms, and we sought to avoid introducing additional equipment into a crowded physical space for the study intervention.

Colorimetric CO_2_ detectors are small, portable, and standardly used to confirm intubation success. Expired CO_2_ is correlated with tidal volume,(24) and colorimetric CO_2_ precedes rise in heart rate among bradycardic infants.(25) Blank et al. targeted UCC to occur >= 60 seconds after colorimetric CO_2_ detection for infants who required respiratory support in a recent feasibility trial.(18) In future studies of infants with CDH, it may be reasonable provide ventilation for a similar duration after CO_2_ detection before UCC.

This single-arm pilot trial was not designed to detect the impact of the study intervention on cardiopulmonary outcomes for infants with CDH. A randomized study powered for relevant outcomes is needed to address this question. There were transient differences in physiologic outcomes such as hemoglobin and blood pressure between trial participants and historical controls. We assessed for echocardiographic evidence of pulmonary hypertension, but these studies- obtained for clinical indications- were performed on average 13 hours after birth and may not have reflected infants’ hemodynamic status immediately after birth. Lefebvre et al. reported that infants treated with intact-cord resuscitation experienced transiently higher systemic blood pressures and blood gas pH values. In the present study, blood gas parameters were similar between participants and historical controls at all assessed time points, and initial pH values were low in both groups. One potential explanation for this is that infants enrolled by Lefebvre et al. had less severe antenatal parameters, with mean O/E LHR of 55%.

We acknowledge study limitations. Neonatal intubations were performed by attending neonatologists. Feasibility outcomes may not generalize to less experienced providers, who typically have lower intubation success rates.(26) In addition, we did not capture granular data on physiologic parameters such as heart rate and oxygen saturation during the initial resuscitation, and non-invasive measures of pulmonary blood flow are not readily available. Study strengths include a prospective trial design with extensive training and oversight by the study team. We used a physiology-based, rather than time-based, approach to UCC.(27) Finally, the trial included infants with a wide spectrum of prognostic CDH parameters, including severely affected infants.

In conclusion, performing intubation and initiating ventilation prior to UCC is safe and feasible among infants with CDH. The impact of this approach on clinically relevant outcomes deserves investigation in a randomized trial.

## Supplementary Material

Supplemental Table

Supplemental video Video (online only): Study intervention performed following a cesarean delivery

## Figures and Tables

**Table 1:** Baseline characteristics of trial participants

Characteristic	Trial Participants (n=20)	Historical Controls (n=19)*
Gestational age, weeks; mean (SD)	38.7 (0.5)	38.5 (0.8)
Birth weight, grams; mean (SD)	3374 (499)	3409 (577)
Male sex	15 (75%)	12 (63%)
Vaginal delivery	8 (40%)	8 (42%)
Left sided defect	15 (75%)	15 (79%)
Any portion of liver in chest	14 (70%)	13 (68%)
O/E LHR %; median (IQR)	30.1 (25.6, 35.9)	34.2 (27.4, 36.7)

IQR: intraquartile range; O/E LHR %: Observed to Expected Lung-to-Head Ratio from anterior-posterior diameter method, by the TOTAL trial calculator for infants with left sided CDH; SD: standard deviation

* one trial participant not matched, due to postnatal diagnosis of major anomaly

**Table 2:** Maternal and infant safety outcomes

Neonatal Outcomes	Trial Participants (n=20)	Historical Controls* (n=19)	P value
Cord Avulsion	0	0	N/A
Chest Compressions	0	0	N/A
Hypothermia on first temperature (<36 Celsius)	3 (15%)	2 (11%)	>0.99
First temperature (Celsius), mean (SD)	36.7 (0.9)	36.8 (0.6)	0.56
Maternal Outcomes			
Estimated blood loss, mL; mean (SD)	583 (230)	528 (210) (n=18)	0.45
Estimated blood loss >500mL	11 (55%)	8/18 (44%)	0.52
Estimated blood loss >1,000mL	0	0	N/A
Therapeutic uterotonics	1 (5%)	2 (11%)	0.61
Wound infection (C/S)	0	0	N/A

C/S: cesarean section; SD: standard deviation

* one trial participant not matched, due to postnatal diagnosis of major anomaly

**Table 3:** Physiologic outcomes

	Trial Participants (n=19)	Historical Controls (n=19)	P Value
Apgar score at 1 minute, median (IQR)	5 (3, 7)	7 (3, 8)	0.51
Apgar score at 5 minutes, median (IQR)	8 (5, 8)	8 (5, 9)	0.72
First Hemoglobin, g/dL; mean (SD)	17.6 (1.3)	16.3 (1.9)	0.02
Mean blood pressure 1 hour after birth; mean (SD)*	51.1 (8.5)	44.3 (6.3)	0.008
First blood gas after birth*			
pH, mean (SD)	7.02 (0.15)	7.03 (0.13)	0.74
CO_2,_ mean (SD)	90 (26)	88 (25)	0.82
Base deficit, mean (SD)	8.9 (3.3)	9.8 (3.8)	0.51
Oxygenation index with first blood gas, median (IQR)	17.5 (12.8, 25.5)	16.3 (12.2, 22.8)	0.74
Vasopressors (first 48 hours)	13 (68%)	16 (84%)	0.45
iNO (first 48 hours)	9 (47%)	11 (58%)	0.52
ECMO (first 7 days)	7 (37%)	4 (21%)	0.48
Mortality (first 7 days)	0	1 (5%)	>0.99

* First blood gas obtained at a mean of 41 minutes after birth. There were no statistically significant differences in blood pressure or blood gas values at 6 hours after birth or beyond

Abbreviations: ECMO: extracorporeal membranous oxygenation; iNO: inhaled nitric oxide; IQR: interquartile range; SD: standard deviation

## “What is already known on this topic”

- Infants with congenital diaphragmatic hernia often experience physiologic compromise immediately after birth
- The traditional approach in the delivery room is immediate cord clamping followed by intubation and ventilation
- Establishing lung aeration through intubation and ventilation prior to umbilical cord clamping may support neonatal transition in these infants

### “What this study adds”

- Intubation and ventilation prior to umbilical cord clamping is safe and feasible among infants with congenital diaphragmatic hernia and may result in short-term physiologic benefit
- The impact of this approach on clinical outcomes requires investigation in a randomized trial

## List of Abbreviations:

CDH: congenital diaphragmatic hernia
ECMO: extracorporeal membrane oxygenation
O/E LHR: observed to expected lung-to-head ratio
UCC: umbilical cord clamping
