# Supplementary material for: Initiating Resuscitation Before Umbilical Cord Clamping in Infants with Congenital Diaphragmatic Hernia: A Pilot Feasibility Trial
Source: Arch Dis Child Fetal Neonatal Ed. Author manuscript; Available in PMC 2021 May 1. (PMC7047568; doi:10.1136/archdischild-2019-317477)
Supplement: Supplemental Table [file NIHMS1060679-supplement-Supplemental_Table.docx]

**Supplemental Table: Echocardiogram assessments**

| **Characteristic** | **Trial Participants (n=18)** | **Historical Control (n=18)** |
| --- | --- | --- |
| Estimated right ventricular pressure above right atrium (mm Hg), median (IQR) | 51 (41, 54)  (n=11) | 49 (39, 50)  (n=11) |
| Direction of shunting at ductus arteriosus |  |  |
| Left to right | 0 | 2 |
| Bidirectional | 14 | 8 |
| Right to left | 3 | 8 |
| No patent ductus arteriosus | 1 | 0 |
| Interventricular septal position |  |  |
| Flattened | 7 | 11 |
| Bowing | 11 | 7 |
| Severity of pulmonary hypertension* |  |  |
| Mild | 0 | 1 |
| Moderate | 6 | 7 |
| Severe | 12 | 10 |

IQR: interquartile range

*Based on the most severe assessment across all three assessments
